# Oxidative Stress in Autism Spectrum Disorder—Current Progress of Mechanisms and Biomarkers

**DOI:** 10.3389/fpsyt.2022.813304

**Published:** 2022-03-01

**Authors:** Xukun Liu, Jing Lin, Huajie Zhang, Naseer Ullah Khan, Jun Zhang, Xiaoxiao Tang, Xueshan Cao, Liming Shen

**Affiliations:** ^1^College of Life Science and Oceanography, Shenzhen University, Shenzhen, China; ^2^Shenzhen-Hong Kong Institute of Brain Science-Shenzhen Fundamental Research Institutions, Shenzhen, China; ^3^Shenzhen Key Laboratory of Marine Biotechnology and Ecology, Shenzhen, China; ^4^Brain Disease and Big Data Research Institute, Shenzhen University, Shenzhen, China

**Keywords:** treatment, oxidative stress, early diagnosis, biomarker, autism spectrum disorder

## Abstract

Autism spectrum disorder (ASD) is a type of neurodevelopmental disorder that has been diagnosed in an increasing number of children around the world. Existing data suggest that early diagnosis and intervention can improve ASD outcomes. However, the causes of ASD remain complex and unclear, and there are currently no clinical biomarkers for autism spectrum disorder. More mechanisms and biomarkers of autism have been found with the development of advanced technology such as mass spectrometry. Many recent studies have found a link between ASD and elevated oxidative stress, which may play a role in its development. ASD is caused by oxidative stress in several ways, including protein post-translational changes (e.g., carbonylation), abnormal metabolism (e.g., lipid peroxidation), and toxic buildup [e.g., reactive oxygen species (ROS)]. To detect elevated oxidative stress in ASD, various biomarkers have been developed and employed. This article summarizes recent studies about the mechanisms and biomarkers of oxidative stress. Potential biomarkers identified in this study could be used for early diagnosis and evaluation of ASD intervention, as well as to inform and target ASD pharmacological or nutritional treatment interventions.

## Introduction

Autism spectrum disorder (ASD) is a type of neurodevelopmental disorder characterized by impaired social communication and interactions, as well as repetitive behavior and limited interests (1). Decades of research have shown that the prevalence of ASD has increased dramatically. According to the Centers for Disease Control and Prevention, one out of every 59 children in the United States is diagnosed with ASD among 8-year-olds in 2018, with boys being four times more likely to be diagnosed than girls (2). ASD's etiology is complex, and it may be due to the interaction of genetic and environmental factors (3, 4). Its development is also heavily influenced by genetic factors (5, 6). Pathogenesis is linked to metabolic disorders, gut microbiota, viral and bacterial infections, chemical influences on the mother's body during pregnancy, as well as neurological and immunological factors (3, 7–9).

There are no clinical biomarkers for ASD because the disorder's etiology and pathogenesis are unknown (3, 10). ASD is diagnosed based on an autism-specific history and clinical observation (3, 10, 11). This could lead to a delay in diagnosis. Although early signs of ASD can be observed and diagnosed as early as 15–18 months of age, the average age of diagnosis is about 4.5 years, and it is not even possible to diagnose ASD before this age (3). Currently, there are no effective pharmaceutical treatments for ASD's fundamental symptoms (12). Early behavioral therapies, on the other hand, have been found to be beneficial in lowering disability and making a significant impact on the outcomes for children with ASD, but they are most effective when started early (13, 14). It has been suggested that interventions initiated before 3 years of age may have a stronger favorable impact than those initiated after the age of five (14). Thus, early diagnosis is essential for ASD, prompting researchers to look for ASD biomarkers. In addition to early diagnosis, reliable ASD biomarker groups are beneficial in clinical practice because they measure the risk of birth in “baby siblings” of children with ASD (15). It also reflects pathogenic processes, assesses treatment and intervention results, and identifies a physiologically homogeneous cohort of ASD patients (16). Moreover, it reveals unknown causes and offers a better knowledge of the disease's underlying pathophysiological processes (17).

However, ASD is a genetically diverse disorder. More than 1,000 genes have been linked to ASD, but none have been found to account for more than 1% of cases (6, 18). Meanwhile, intellectual disability, trouble coordinating movement, sleep difficulties, seizures, and gastrointestinal (GI) issues are also common comorbidities associated with ASD (19). Therefore, identifying biomarkers for ASD has been challenging. Despite this, the evidence suggests that immunological dysregulation, inflammation, oxidative stress, mitochondrial dysfunction, and excitotoxicity are key components in ASD pathogenesis (3, 10, 20, 21). The biomarker related to them have been detected in the blood and urine, and these abnormalities have also been observed in the brain of individuals with ASD, indicating that they could be used to reduce diagnostic heterogeneity and enhance treatment response prediction. So far, many studies have reported increased oxidative stress in individuals with ASD, including decreased enzymatic antioxidants, and increased DNA, lipid, and protein oxidation products both in the brain and peripheral circulation (22–28). Increased oxidative stress markers have been found in peripheral body fluids and have been linked to ASD severity (29).

This article reviews the current state of research on oxidative stress in ASDs, focusing on the mechanism of oxidative stress, biological analysis of oxidative stress biomarkers, and antioxidant-based therapy methods. The literature focused on the last 10 years and was collected from PubMed, Web of Science, and Google Scholar.

## Oxidative Stress and ASD

The concept of oxidative stress was initially introduced in 1985 with the publication of the book “Oxidative Stress” (30). Reduction–oxidation (redox) reaction is a type of indispensable reaction in the cellular physiological process of cells, during which ROS are generated. ROS is typically produced either intentionally (to kill invading pathogens or as intermediates in enzymatic reactions, etc.) or accidentally (*via* electron leakage from electron transport chains, metabolism of drugs, exposure to chemicals, pollutants, and radiation, etc.) during normal physiological processes of cells. The sources of ROS contain many enzymes. Nicotinamide adenine dinucleotide phosphate oxidase (NOX) isoforms are major sources for endogenous ROS, multifarious NOX isoforms are localized to various cellular membranes and involved in many physiological or pathological events (31, 32). Myeloperoxidase (MPO) is primarily located in immune cells and plays an important role in our immune system, which produces some ROS, particularly hypochlorous acid (HOCl) to kill invading pathogens (33). NO synthases (NOSs) are the most important NO source in both physiological and pathological conditions (34). Interestingly, low concentrations of NO generated by neuronal NOS or endothelial NOS have a physiological neuroprotective function and are involved in signaling pathway, while higher concentrations of NO synthesized by inducible NOS (iNOS) are neurotoxic (35, 36). Beside the sources of ROS mentioned above, there are also many ROS-generating enzymes include succinate dehydrogenase (SDH) (37), dihydroorotate dehydrogenase (DHOH) (38), mitochondrial glycerol-1-phosphate dehydrogenase (mGPDH) (39), cytochrome b5 reductase (40), monoamine oxidases (MAOs) (41), aconitase (ACO) (42), xanthine oxidoreductase (XOR) (43), alpha-Ketoglutarate dehydrogenase complex (KGDHC) (44), and so on. Some of these enzymes were shown to produce ROS at appreciable rates in studies with either isolated enzymes or mitochondria (45). Additionally, it should be noted that ROS is a general term but not some specific molecule (46–48). It contains a group of molecules that come from molecular oxygen, such as superoxide (O${}_{2}^{•-}$), hydrogen peroxide (H_2_O${}_{2}^{•}$), hydroxyl radical (OH^•−^), and peroxyl radical (RO${}_{2}^{•-}$), and the chemical reactivity of each ROS molecule is quite different (46, 47, 49).

ROS are eliminated by the antioxidant defense of cells in normal physiological processes, and the body is in a state of physiological balance. This balance, however, will be disrupted if there is an increase in ROS production or a decrease in cell antioxidant capacity, resulting in oxidative stress (46, 47). When there is mild oxidative stress, a low level of ROS stimulates the cellular defense mechanism to produce a proper response to ROS, while ROS can also induce cell apoptosis as a signal molecule. This phenomenon is known as “eustress,” and it is beneficial to the maintenance of cellular ROS defense and tissue renewal (48, 50, 51). When cells are subjected to severe oxidative stress, ROS that is out of balance with antioxidant capacity damage biomolecules such as proteins, lipids, and DNA, as well as some biological structures such as bio-membrane structure. This is known as “distress.”

As the energy factories of cells, mitochondria are the main sites for the generation of ROS, in which the electron transport chain (ETC) is a prime source for ROS (38). Both endogenous and exogenous oxidative stress can cause a deficit in mitochondrial ETC complexes, resulting in mitochondrial dysfunction. Dysfunctional mitochondria produce more ROS, which can further impair mitochondrial function. This is a vicious cycle in which more severe oxidative stress and mitochondrial dysfunction occur if ROS is not eliminated promptly due to decreased antioxidant capacity. Glutathione (GSH) has been shown in numerous studies to play an important role in mitochondrial ROS elimination (24, 26, 52, 53).

**Figure 1:**
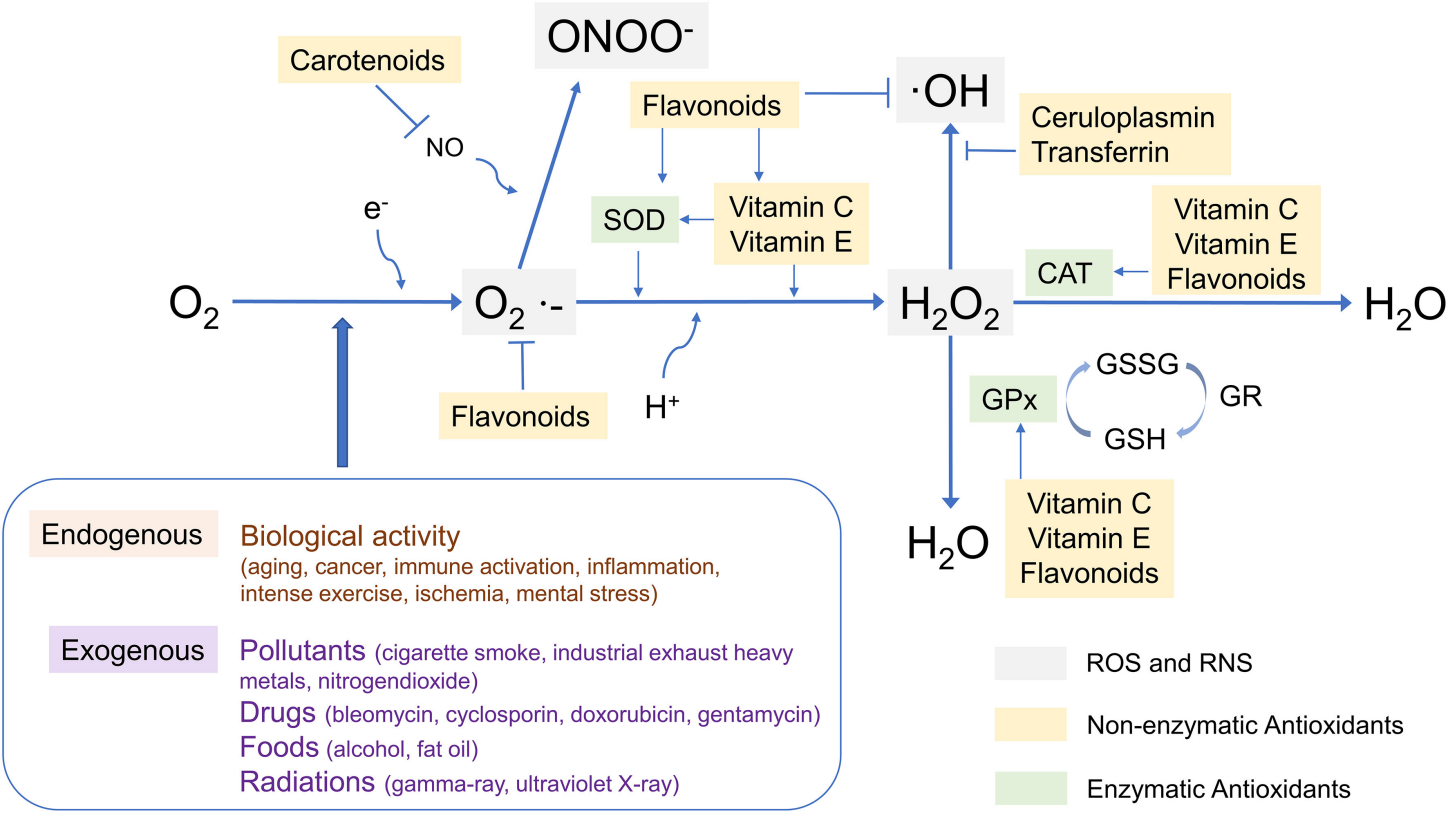
The interaction between some common antioxidants and ROS. SOD, superoxide dismutase; CAT, catalase; GPx, glutathione peroxidase; O2·−, superoxide; ·OH, hydroxyl radical; ONOO−, Peroxynitrite; GSSG, glutathione oxidized; GSH, glutathione; GR, gluathione reductase.

The antioxidant capacity of various cellular defense systems is based on enzymatic antioxidants such as superoxide dismutase (SOD), catalase (CAT) and glutathione peroxidase (GSH-Px), and non-enzymatic antioxidants such as ascorbic acid (vitamins C), uric acid, tocopherol (vitamins E), quinols, carotenoids, and polyphenols. The interaction between some common antioxidants and ROS has been shown in Figure 1 (54–65). It is worth noting that some enzymatic antioxidants have multiple isoforms, and the different isoforms have different functions, such as SOD (66), GSH-Px (67), etc. Furthermore, as a complement to defense systems, some repair systems, such as methionine sulfoxide reductases, disulfide reductases/isomerases, phospholipases, and DNA repair enzymes, will repair structures and biomolecules that have been damaged or modified by residual ROS. As a result, when cells are subjected to mild oxidative stress, these mechanisms can effectively protect them from oxidative damage (46–48). Although there are numerous mechanisms in cells to combat oxidative stress, numerous studies show that oxidative damage to biological structures and biomolecules continues to accumulate in cells in many related diseases, including ASD (46, 47, 68–72).

The human brain is the largest oxygen-consuming organ in the body. It only accounts for 2% of the body mass but consumes 20% of the oxygen. It has a high content of oxidizable polyunsaturated fatty acids as well as redox-active metals (copper and iron). As a result, the human brain is particularly vulnerable to oxidative stress (47, 73–75). Children are more vulnerable than adults to oxidative stress because of their naturally low glutathione levels from conception to infancy (72). In the brains of children with ASD, low levels of mitochondrial glutathione and mitochondrial dysfunction have been reported (24, 26, 52, 53). In particular, increased oxidative stress has been observed in the brains of children with ASD (24). Oxidative stress causes oxidative damage to lipids, proteins, and DNA in cells. It makes a variety of reversible and irreversible damages in ASD which mainly involves various post-translational modifications of proteins such as 3-nitrotyrosine (3NT) and protein carbonyl formation, abnormal metabolism such as lipid peroxidation, and accumulation of toxic such as ROS. The relationship between oxidative stress and ASD has recently been thoroughly reviewed (76). Many markers of oxidative stress, such as lipid peroxide (LOOH) (77), malondialdehyde (MDA) (78), a marker of oxidative DNA damage 8-hydroxy-2'-deoxyguanosine (8-OH-dG) (24), protein carbonyl (28, 79), and 3-nitrotyrosine (3-NT), are elevated in children with ASD. The increased oxidative stress markers have been observed to be correlated with ASD severity (29).

Furthermore, several studies have shown that oxidative stress causes an inflammatory response as an upstream component in the signaling cascade (80, 81). ASD patients have been shown to have systemic immunological abnormalities as well as an inflammatory response (82, 83). In fact, oxidative stress is often detected alongside inflammation in the brains of people with ASD, and some studies have demonstrated a link between the two in specific brain regions associated with ASD (24, 84, 85). Even though it is difficult to know whether the connection is unique to specific brain regions or not due to the limitations of the brain tissue sample, this has revealed more about the role of oxidative stress in the etiology of ASD. Other studies in peripheral blood cells have found evidence of inflammation and oxidative stress in a variety of cell types, including T cells (86, 87), B cells (88), monocytes (89, 90), neutrophils (90), and lymphocytes (91). In these studies, *in vitro* induction experiments were also used to demonstrate the link between inflammation and oxidative stress in peripheral cells. Peripheral cells may be useful in studying systemic neurochemical changes in ASD.

**Figure 2:**
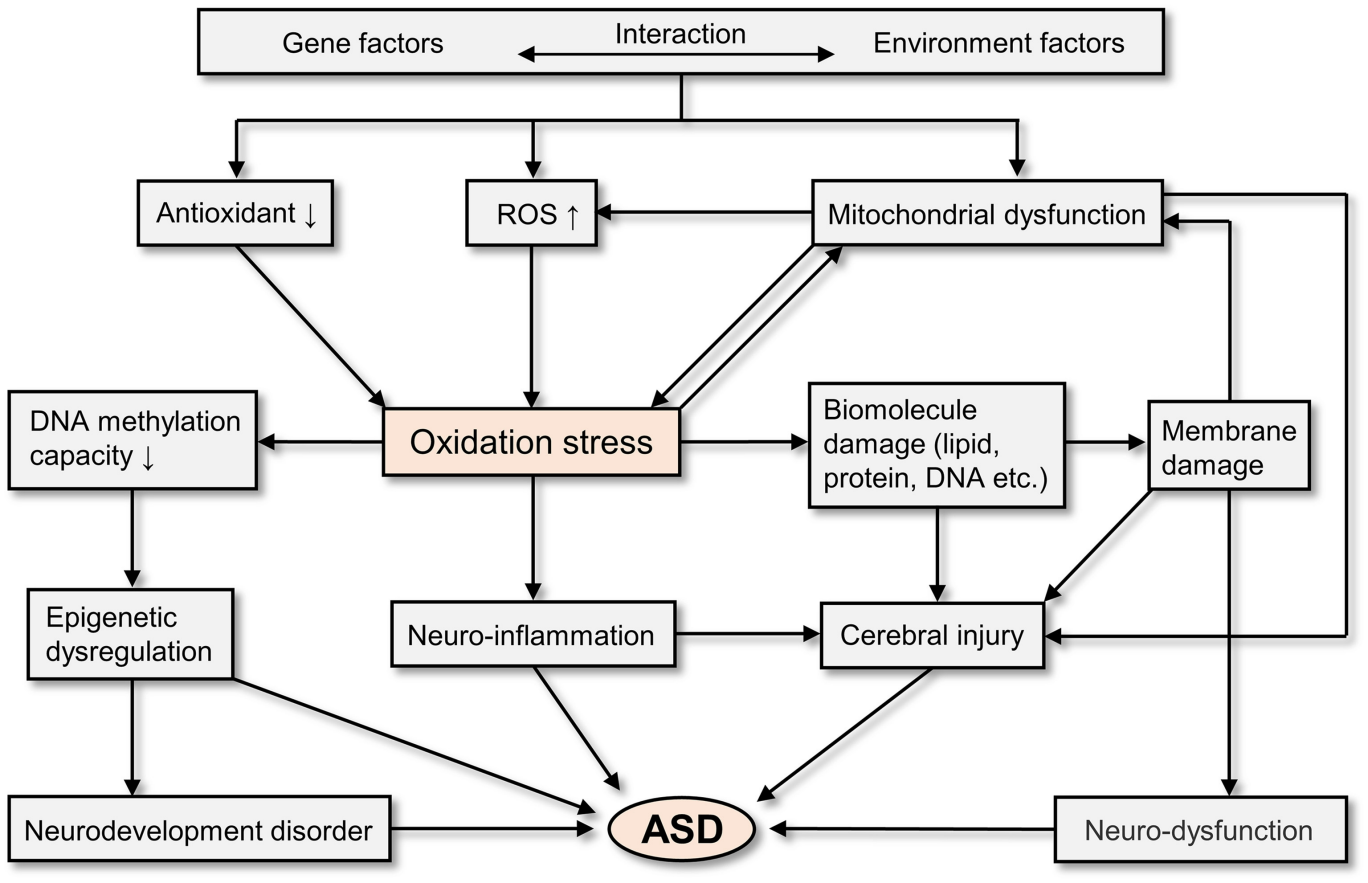
The potential mechanisms of oxidative stress in the brain of ASD patients.

In general, oxidative stress is involved in the pathogenesis of ASD. As a result of the interaction of genetic and environmental factors, people with ASD have excessive ROS production, decreased antioxidant capacity, and mitochondrial dysfunction (55). All of these physiological abnormalities have the potential to cause oxidative stress (55, 92, 93). And oxidative stress can cause epigenetic dysregulation (55, 93), neurodevelopment disorder (94), neuro-inflammation (95), cerebral injury (55, 92, 95), and neuro-dysfunction (55, 92, 95), which finally leads to ASD (94–96). Figure 2 depicts the potential mechanisms of oxidative stress in the pathogenesis of ASD.

## Biomarkers of Oxidative Stress

The studies of potential oxidative stress biomarkers for ASD in the past 10 years are shown in Table 1. It focuses on proteins and metabolites related to oxidative stress in peripheral body fluids such as blood, urine, and saliva. These potential biomarkers include enzymatic antioxidants, non-enzymatic antioxidants, proteins, and lipids damaged by oxidation (28, 29, 97–140). It is critical to verify whether the changes reported in different research are consistent as a potential biomarker. Individual variances must be adapted by effective biomarkers, especially in the case of disease as heterogeneous. Most of these potential biomarker changes in ASD patients are consistent without dissenting reports. Here, we will focus on a few classic oxidative stress biomarkers related to ASD and introduce them according to their classification.

**Table 1:** The studies of potential oxidative stress biomarkers for ASD in the past 10 years.

**References**	**Method**	**Sample size (ASD/control)**	**Detail (increased or decreased, compared with the control group)**	**Sample type**
Meyyazhagan et al. (97)	ELISA, ESA coulometric electrode array system	98/98	Increased: serotonin, γ-Aminobutyric acid, homocysteine Decreased: ceruloplasmin, transferrin, pyruvate kinase and hexokinase	Blood
El-Ansary et al. (98)	Biochemical analyses	13/24	Increased: coenzyme Q10, caspase 7, melatonin Decrease: glutathione	Plasma
Hamed et al. (99)	ELISA	38/32	Increased: TGFβ2, Heat shock protein 70 Decreased: hematopoietic prostaglandin D2 synthase	Blood
Hassan et al. (100)	Biochemical analyses	73/73	Increased: L-carnitine	Blood
Faber et al. (101)	Isotope dilution mass spectrometry (IDMS), speciated isotope dilution mass spectrometry (SIDMS), LC-MS/MS	30/30	Increased: glutathione, concentrations of oxidized glutathione Decreased: total/oxidized glutathione ratio	Blood
El-Ansary et al. (102)	Biochemical analyses	27/27	Increased: 8-Hydroxy-deoxyguanosine Decreased: 25-Hydroxyvitamin D2	Blood
Howsmon et al. (103)	Biochemical analyses	83/76	Increased: oxidized glutathione, nitro-tyrosine Decreased: glutathione, tyrosine	Blood
Meguid et al. (104)	LC-MS, quantitative reverse-transcription PCR (qRT-PCR)	80/60	Decreased: GCLM, SOD2, NCF2, PRNP, and PTGS2	Blood
Khemakhem et al. (105)	ELISA	41/41	Increased: pyruvate, lactate dehydrogenase, creatine kinase, complex 1, glutathione S-transferase, coenzyme Q10, caspase 7, melatonin Decreased: glutathione	Plasma
El-Ansary et al. (106)	Biochemical analyses	30/30	Increased: creatine kinase, ectonucleotidase (ATPase), ectonucleotidase (ADPase), Na+/K+ (ATPase), lactate, glutathione peroxidase, superoxide dismutase, lipid peroxides Decreased: inorganic phosphate, ATP, glutathione, vitamin C (oxidized), vitamin E	Plasma
Signorini et al. (107)	GC-MS/MS,	61/61	Increased: plasma 10-F4t-NeuroP content Decreased: 4-F4t-NeuroP levels	Plasma
Feng et al. (28)	2D-Oxyblot, Western blot, Immunoprecipitation	15/15	Increased: complement component C8 alpha chain, immunoglobulin kappa chain C	Plasma
Metwally et al. (108)	ELISA	49/40	Increased: bisphenol A, 8-Hydroxydeoxyguanosine	Serum
El-Ansary (109)	Biochemical analyses	20/20	Increased: glutamic, thioredoxin I, thioredoxin reductase, peroxiredoxin I, peroxiredoxin III Decreased: glutathione, glutamate dehydrogenase	Blood
Qasem et al. (110)	Biochemical analysis	44/40	Increase: 8-isoprostane, cysteinyl leukotrienes	Plasma
Cortelazzo et al. (111)	LC-MS/MS, Biochemical analyses	30/30	Increased: triglycerides, total cholesterol, eosinophil counts, alpha-2-macroglobulin, alpha-1-antitrypsin, haptoglobin, serum transferrin, pre-albumin, apolipoprotein J, 4HNE, fibrinogen beta chain, serum albumin, immunoglobulin alpha-1 chain, immunoglobulin gamma heavy chains	Plasma
Ciccoli et al. (112)	GC / MS	15(the predominant ASDs phenotype)/15(non-autistic neurodevelopmental disorders)/15(healthy control)	Decreased: β-actin	Blood
Ghezzo et al. (29)	Biochemical analyses, gas chromatograph	21/20	Increased: thiobarbituric acid reactive substances (TBARS), DHA-ω6/ω3 ratio, 1-6-phenyl-1,3,5-hexatriene (DPH), 1-(4-trimethylammoniophenyl)-6-phenyl-1,3,5-hexatriene (TMA-DPH) Decreased: Na/K ATPase activity, erythrocyte membrane fluidity, EPA, and DHA-ω3,	Blood
Gorrindo et al. (113)	GC / MS	27(ASD and GID)/29(ASD without GID)/21(GID without ASD)/10(control)	Increased: F2t-isoprostanes	Plasma
Frye et al. (114)	High-performance liquid chromatography, electrochemical detection	18(ASD with MD)/18(ASD without MD)/18(control)	Increased: 3-chlorotyrosine Decreased: free reduced glutathione, free reduced glutathione/oxidized glutathione ratio	Plasma
El-Ansary and Al-Ayadhi (115)	ELISA	20/19	Increased: prostaglandin E2, leukotrienes, isoprostanes	Plasma
Melnyk et al. (116)	Electrochemical detection, Biochemical analyses	68(ASD)/54(CON)/40(ASD Sibling)	Decreased: methionine, S-adenosylmethionine, adenosine, 5-methyl-cytosine, oxidized glutathione, glutathione Increased (different with sibling but not control): increase in leukocyte DNA 8-oxo-deoxyguanosine	Plasma
Essa et al. (117)	Biochemical and data analysis	20/20	Decreased: ceruloplasmin, transferrin	Plasma
Lakshmi Priya and Geetha (118)	SDS-PAGE, Western blot	45/45	Decreased: TBARS, glutathione, vitamin A, vitamin C, superoxide dismutase	Blood
Essa et al. (119)	Biochemical analyses	19/19	Increased: the levels of NO, malondialdehyde, protein carbonyl, and lactate to pyruvate ratio	Blood
Rose et al. (120)	PCR, Biochemical analyses, Seahorse Extracellular Flux	43/41	Increased: glutathione Decreased: oxidized glutathione disulfide	Blood
Al-Yafee et al. (121)	Biochemical analyses	20/20	Increased: thioredoxin, thioredoxin reductase, peroxiredoxin 1, peroxiredoxin 3 Decreased: reduced glutathione, total glutathione, GSH/GSSG and activity levels of GST	Plasma
Adams et al. (122)	LC-MS/MS	55/44	Increase: adenosine, uridine Decrease: S-adenosylmethionine	Plasma
El-Ansary et al. (123)	Gas chromatograph	26/26	Increased: acetic, valeric, hexanoic, stearidonic Decreased: propionic, butyric, caprylic, decanoic, lauric, palmitic, stearic, arachidic, a-linolenic, eicosapentaenoic, docosahexaenoic, linoleic, arachidonic, oleic, elaidic	Plasma
El-Ansary et al. (124)	Biochemical analyses	25/16	Increased: acetic, valeric, hexanoic, stearidonic Decreased: propionic, butyric, caprylic, decanoic, lauric, palmitic, stearic, arachidic, a-linolenic, eicosapentaenoic, docosahexaenoic, linoleic, arachidonic, oleic, elaidic	Plasma
Ali et al. (125)	Enzyme immunoassay, automated random-access immune-assay system	40/40	Increased: Hcy levels Decreased: folate, vitamin B12	Serum
AL-ayadhi and Mostafa (126)	ELISA	42/42	Increased: osteopontin	Serum
Khakzad et al. (127)	High-sensitivity CRP test	39/30	Increased: hs-CRP concentrations	Serum
Meguid et al. (128)	Biochemical analyses	20/25	Increased: malondialdehyde Decreased: glutathione, glutathione peroxidase	Blood
Ming et al. (129)	PCR	103/0	Significant transmission disequilibrium was found in the overall transmission of the human glutathione peroxidase (GPX1) polyalanine repeat (ALA5, ALA6, and ALA7). The ALA6 allele was under transmitted.	Blood
Osredkar et al. (130)	ELISA	139/47	Decreased: 8-hydroxydeoxyguanosine	Urine
Yui et al. (131)	ELISA, SOD Assay Kit	20/11	Increased: hexanoyl-lysine	Urine
Puig-Alcaraz et al. (132)	LC-MS, Biochemical analyses	35/34	Increased: homocysteine	Urine
Ranjbar et al. (133)	Biochemical analyses	29/24	Increased: catalase activity Decreased: total antioxidant concentration, total thiol molecules	Urine
Kałuzna-Czaplińska et al. (134)	GC / MS	34/21	Increased: homocysteine	Urine
Kałuzna-Czaplińska (135)	GC / MS	35/36	Increased: 2-oxoglutaric acid, isocitric acid, citric acid, 4-hydroxybenzoic acid, 4-hydroxyphenylacetic acid, hippuric acid, adipic acid, suberic acid, arabinitol Decreased: tryptophan	Urine
Damodaran and Arumugam (136)	UV spectrophotometric, Biochemical analyses	45/50	Increased: lipid peroxides, lipid hydroperoxides, protein carbonyl, total peroxides, uric acid/creatinine, malondialdehyde, 4-hydroxynonenal Decreased: protein sulfhydryl, non-protein sulfhydryl, and total sulfhydryl, level of creatinine excreted	Urine
Youn et al. (2010) (137)	LC-MS/MS	65/9	Increased: proporphyrins, pentacarboxyporphyrin, precoproporphyrin, coproporphyrins, and total porphyrins	Urine
Ngounou Wetie et al. (138)	Two-dimensional PAGE, LC-MS/MS, HPLC	6/6	Increased: proto-oncogene FRAT1, Ig alpha-1 chain C region, immunoglobulin heavy chain constant region alpha-2 subunit, V-type proton ATPase subunit C 1, Kinesin family member 14, Integrin alpha 6 subunit, growth hormone regulated TBC protein 1, parotid secretory protein, Prolactin-inducible protein precursor, Mucin-16, Ca binding protein MRP14 Decreased: alpha-amylase, CREB-binding protein, p532, Transferrin variant, Protein-L-isoaspartate O-methyltransferase domain-containing protein 1 isoform 3, Chain A of Human Pancreatic Alpha-Amylase In Complex With Myricetin, V-type proton ATPase subunit C 1, Ig J-chain, Zn alpha2 glycoprotein, Glutamate-rich protein 6B, Immunoglobulin heavy chain variable region, Albumin protein, Sperm activating protein subunit I-Apo A1-SPAP-subunit I, Zymogen granule protein 16 homolog B precursor, Putative lipocalin 1-like protein 1,cystatin D and plasminogen	Saliva
Anwar et al. (139)	LC-MS/MS	38/31	Increased in plasma: Nε-carboxymethyl-lysine, Nω-carboxymethylarginine, dityrosine Increased in urine: alpha-aminoadipic semialdehyde, glutamic semialdehyde, asn, pro, ser, and val Renal clearance of carboxymethylarginine, glucosepane, dityrosine, arg, glu, leu, phe, and thr were decreased and renal clearance of N-formylkynurenine and trp were increased in children with ASD, with respect to healthy controls.	Plasma and urine
Yenkoyan et al. (140)	LC-MS/MS, ICP-MS, flow cytometry	10/10	Increased: 8-hydroxy-2'-deoxyguanosine Decreased: superoxide dismutase	Blood and urine

### Blood-Based Biomarker

#### GSH, GSSG, and GSH/GSSG

Genetic variations in glutathione-related pathways have been observed in ASD (141–144) and have been correlated to ASD behaviors in some studies (145, 146). GSH has been reported as a biomarker of ASD oxidative stress in numerous studies, as shown in Table 1. The levels of GSH in the blood of autistic patients have been reported to be variable. Some studies have found that GSH levels are elevated when compared to healthy controls, while other studies have found lower levels (101). However, a recent meta-analysis found that GSH and total glutathione (tGSH) levels in the blood are lower in people with ASD compared to controls (147).

GSH is an important antioxidant in the human body that protects against oxidative stress. It has the ability to detoxify cytotoxic molecules. Lower GSH levels were found to be associated with the severity of ASD in a previous study (148). Toxic metals are one of the environmental factors that contribute to ASD. They can cause oxidative stress, which can lead to ASD (4, 149). In this case, it will expend a significant amount of GSH (150, 151). This may be one of the reasons for the disparities in the results of different studies. In general, as oxidative stress increases, it appears that GSH levels will decrease as consumption exceeds production. However, there is a compensation effect on the human body. In order to resist the increased oxidative stress, the production of GSH may be increased (152–154). Therefore, the diversity of GSH functions and individual differences are responsible for the difference in GSH levels in ASD patients. GSH, on the other hand, is known to be converted into oxidized glutathione (GSSG) by glutathione peroxidase and reduced back to GSH by glutathione reductase in the human body. The increased GSH consumption caused by oxidative stress will disrupt this dynamic equilibrium of GSH and GSSH (152–154). Therefore, many studies have detected not only the level of GSH in the sample but also the GSH/GSSG ratio. Interestingly, the rising GSH/GSSG ratio is a consistent result in all related studies (101, 103, 105, 114, 121), indicating that it is a good indicator of oxidative stress in the human body. This is consistent with the findings of a meta-analysis of oxidative stress marker abnormalities in children with ASD (147). In this meta-analysis, GSSG was found to be increased in autistic children, while tGSH/GSSG was decreased (147).

Taken together, blood glutathione metabolism markers are one of the important ASD oxidative stress markers. In different studies, it usually demonstrated constant and high significant differences between ASD children and controls (147). Besides, a postmortem study showed that GSH and GSH/GSSG were significantly decreased in the brains of ASD patients relative to controls (24). These glutathione metabolism markers may show parallel changes between the central and peripheral nervous systems in ASD.

#### Homocysteine and Vitamin B6, B9, and B12

Hcy is a non-protein amino acid derived from the methionine cycle that is required for activated methyl transfer and the trans-sulfuration pathway (155). Previous research on Hcy levels in the blood of autistic children has yielded conflicting results (156, 157). Some studies showed a significant decrease (142, 158), while others found no difference (159, 160). However, in accordance with two recent meta-analyses (147, 155), some blood studies have consistently found that increased levels of Hcy were found in the blood of ASD patients (Table 1) (97, 161).

Hcy is located at the intersection of the methionine cycle and trans-sulfuration pathway. The methionine cycle is responsible for the production of the universal methyl donor S-adenosylmethionine (SAM), which is used in a variety of methyl transfer reactions. The trans-sulfuration pathway is related to the synthesis of GSH (155). Changes in Hcy levels may have an impact on these two metabolic pathways. Impairment of methionine circulation, abnormal trans-sulfur metabolism (52, 142, 158), and alterations in DNA methylation (162) have been shown to be associated with the development of ASD (155). The concentration of SAM in children with ASD was higher than that in the healthy controls, while SAM/S-adenosylhomocysteine (SAH) was significantly lower (147).

Various B vitamins such as B6 (pyridoxine), B9 (folic acid), and B12 (cobalamin) play important roles in the development, differentiation, and functioning of the central nervous system. They are involved in the methionine-homocysteine pathway (163). The levels of vitamin B9 and B12 in the blood of ASD children were significantly lower than those in the control group (147, 164, 165). Their deficiency causes a decrease in homocysteine re-methylation, resulting in an increase in homocysteine levels (166). A lack of vitamin B12 may result in DNA hypomethylation, affecting the development of the central nervous system (167). Vitamin B deficiency can be caused by a lack of nutrients, poor absorption, or intestinal disorders. The gut microbiota is essential for digestion because it synthesizes essential dietary vitamins and cofactors such as vitamin B, riboflavin, thiamine, and folic acid (168). Folate deficiency and high Hcy levels are especially harmful to the neurological system (169, 170) because Hcy has neurotoxic characteristics (155).

**Figure 3:**
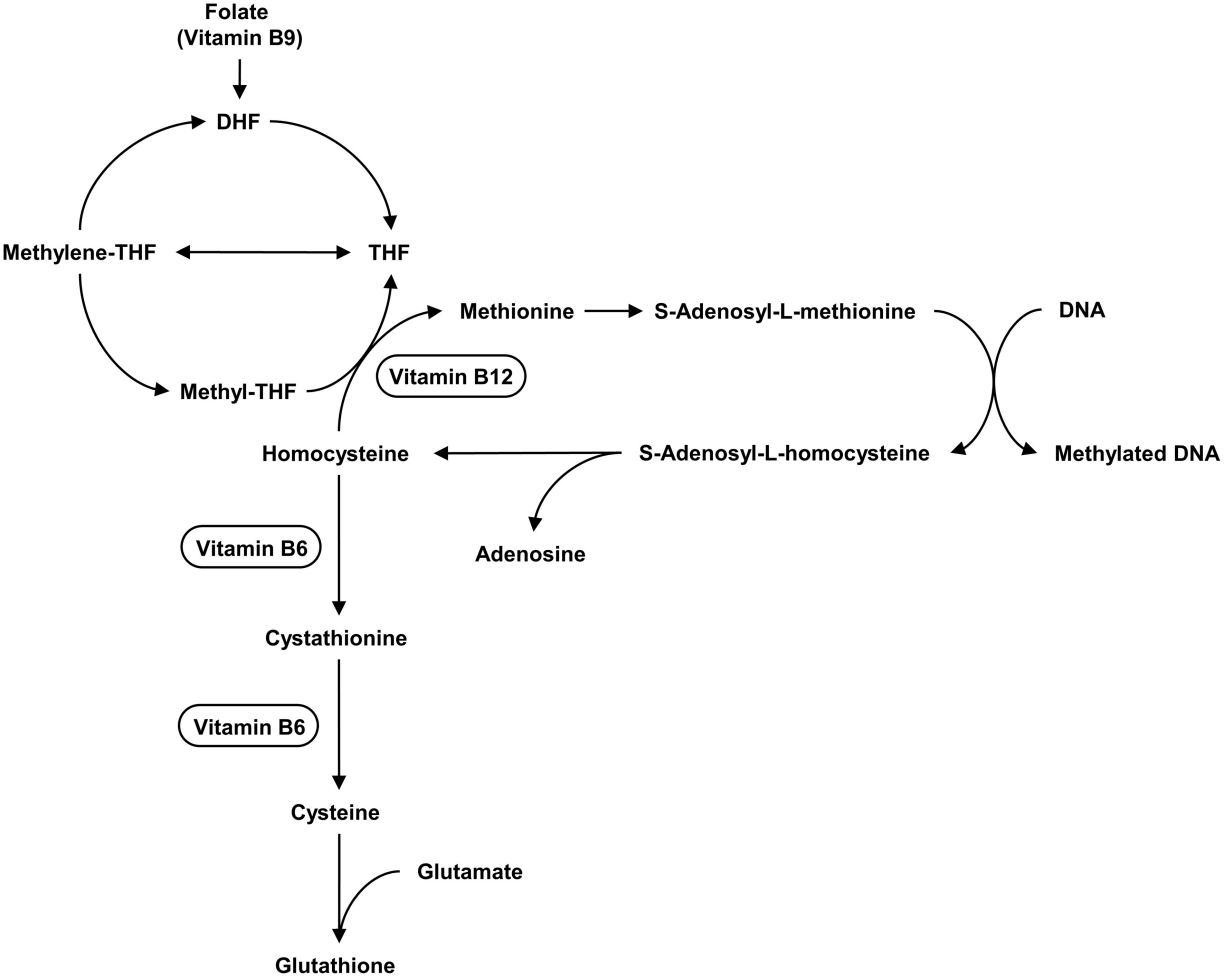
The metabolism of Hcy and the relationship between vitamins and this metabolism. Hcy is located at the intersection of the methionine cycle and the transsulfuration pathway. The methionine cycle is responsible for producing the universal methyl donor SAM, which is used in various methyl transfer reactions. The transsulfuration pathway is related to the synthesis of GSH. Vitamin B12, Vitamin B6 act as cofactors in the enzymatic reactions in cycle.

Together, Hcy, vitamin B6, B9, and B12 may be associated with the pathophysiology of ASD. Figure 3 depicts the relationship between vitamins and the metabolism of Hcy. Children with ASD may have genetic and physiological disorders, poor lifestyle choices (including dietary habits), and a variety of pathological conditions, therefore monitoring their levels is important. Hcy studies are, however, heterogeneous, and more research is needed (155).

#### MDA and 4-Hydroxynonenal

Lipid peroxidation is an important part of oxidative stress and can be explained as a process in which ROS free radicals attack lipids containing carbon-carbon double bonds, especially polyunsaturated fatty acids (PUFAs) (171). Lipid peroxides are the main products in this process.

The phospholipid bilayer is primarily composed of PUFAs. When exposed to oxidative stress, ROS will constantly attack membrane lipids until they are depleted. Membrane lipid peroxidation results in a number of membrane changes, including increased membrane rigidity, decreased activity of membrane-bound enzymes, altered activity of membrane receptors, and altered permeability (172, 173). On the other hand, some PUFAs such as arachidonic acid (AA) can also be oxidized to various signaling molecules by specific enzymes like lipoxygenases (LO) and thus play a role in the regulation of many important physiological functions (174, 175). These signaling lipids include diacylglycerol (176–178), inositol phosphates (179–181), prostaglandins (182–184), and steroid hormones (185), etc. However, whether lipids are oxidized by enzyme action or by ROS attack, the process results in a variety of classic biomarkers of lipid peroxidation such as MDA, 4-HNE, and F2-isoprostane, etc. A study reported that MDA and 4-HNE levels were higher in the frontal brain of ASD patients (186). This suggests that lipid peroxidation occurs in ASD patients' brains and may be related to the pathological process of ASD self-enhancement.

Despite the significant investigation, MDA has only been recognized as a signaling molecule in a few studies, such as regulation of islet glucose-stimulated insulin secretion (GSIS) (187) and gene expression of specificity protein-1 (*Sp1*) in hepatic stellate cells (188). MDA is known for its cytotoxicity, which occurs when it forms adducts with proteins, notably membrane proteins (189–192). MDA is also involved in DNA damage and mutation (193, 194), which leads to cell cycle cessation (195). In several studies of children with ASD, increased MDA content in the blood has been observed as a typical sign of lipid peroxidation (23, 119, 128). In one study, however, MDA levels in the blood of ASD patients did not decrease significantly (196). In fact, this discrepancy could be due to the measurement method. Thiobarbituric acid reactive substances (TBARS) is a well-known MDA measurement method based on MDA and thiobarbituric acid reaction (TBA) (197). However, this method is non-specific because many carbonyl compounds such as some oxidized unsaturated fatty acids have been shown to react with TBA and interfere with MDA measurement (198). In addition, according to the biochemical properties of MDA, biological MDA will exist in two forms: free MDA and adducted MDA with proteins, nucleic acids, lipoproteins, and amino acids (199). Some researchers attempted to detect MDA using liquid chromatography-mass spectrometry (LC-MS) and gas chromatography-mass spectrometry (GC-MS), which showed to be more specific and sensitive than TBARS analysis, as well as effective for both free and adducted MDA (200, 201). Except in blood investigations, amino acid adducts of MDA, such as N-epsilon-(2-propenal) lysine, N-α-acetyl-(epsilon)-(2-propenal) lysine, N-(2-propenal) serine, and N-(2-propenal) ethanolamine (202–205) have been found in urine by mass spectrometry.

4-HNE is also a lipid peroxidation end product and one of the most cytotoxic products. Once produced in cells, its elimination depends mainly on the action of antioxidants like GSH. It is intriguing that 4-HNE, as a signaling molecule, can regulate the expression of many transcription factors while enhancing the antioxidant mechanism of cells. These transcription factors include nuclear factor erythroid 2-related factor 2 (Nrf2) (206, 207), activating protein-1 (AP-1) (208), and peroxisome proliferator-activated receptors (PPAR) (209, 210), etc. At the same time, 4-HNE is highly cytotoxic causing protein and DNA damage (199, 211–213), affecting autophagy (214, 215), and inducing cell apoptosis (216). Furthermore, high concentrations of 4-HNE can cause cell necrosis (217). Similar to the biochemical properties of MDA, biological 4-HNE also exists in two forms, including free 4-HNE or adducted 4-HNE with proteins, nucleic acids, lipoproteins, and amino acids (152). Based on the HPLC-based free 4-HNE measurement method, 4-HNE absorbs (197) in the UV range (220–223 nm). Other more specific and sensitive probes that are widely used are aldehyde reaction probes such as 2,4-dinitrophenylhydrazine (DNPH) and 1,3-cyclohexanedione (CHD) (218). UV spectrophotometry was used in a study to detect significant increases of 4-HNE in the urine of children with ASD (136). These methods, like MDA, are non-specific because they cannot distinguish 4-HNE from other aldehydes. The early measuring method of adducted 4-HNE, on the other hand, is an immunoassay, which relies on antibodies specific for 4-HNE bound to proteins or other biomolecules (219, 220). Using a Western blot assay, a study found a significantly higher level of 4-HNE protein adducts (4-HNE PAs) in the plasma of children with ASD (221). Many specific detection methods for 4-HNE, including adducted 4-HNE based on LC-MS or GC-MS, have been developed as a result of the advancement of mass spectrometry technology, and are now widely used in the detection of biomarkers of various oxidative stress diseases (222). Unfortunately, few studies have focused on 4-HNE as a biomarker in ASD. A study on the brain tissue from ASD patients showed that cellular stress and apoptosis caused by 4-HNE in the brain may contribute to the pathogenesis of ASD (223), implying that 4-HNE is a worthy research direction.

In addition to MDA and 4-HNE, the most prominent markers of lipid peroxidation, such as isoprostanes, have been established as biomarkers and have received extensive attention. Multiple studies have discovered elevated levels of isoprostane in blood samples from children with ASD, as indicated in Table 1 (65, 68, 70). More study is needed at this time on the types of biomarkers and application methods that can more correctly identify the extent of lipid peroxidation in patients.

### Urine Based Biomarker

As shown in Table 1, there have been few biomarker studies of oxidative stress in ASD utilizing urine samples. Despite the fact that blood has a more complicated composition, urine biomarker studies have lagged behind those in the blood (224). This could be related to the fact that urine biomarkers are limited. Gender, age, collection time, dietary choices, and kidney injury are only a few of the factors that produce changes in urine components. Because urine is more unstable than blood, reliable biomarkers must be revealed before it can be discovered. Furthermore, several high-abundance proteins in urine, such as uromodulin, albumin, and immunoglobulin, might obstruct the detection of low-abundance proteins. As a result, enlarging low abundance urine proteins or eliminating high abundance urinary proteins should be considered (225).

Although there are certain limitations to detecting biomarkers in urine, urinary biomarkers still offer great potential and advantages. Urine is one of the body's principal excretory systems, containing a variety of proteins and metabolites, many of which are well-described in both normal and pathological conditions (226). Urine collection is safer, more convenient, and yields a bigger sample volume when compared to other peripheral bodily fluids. Because urine generation is linked to plasma filtration and selective reabsorption, changes in urine components can signal not only the presence of disorders like diabetes and kidney disease but also the presence of changes in blood components.

Interestingly, as indicated in Table 1, oxidative stress biomarkers have been found in the blood and urine of ASD patients in several studies (139, 140). These investigations focus on the antioxidant capacity of blood and urine, as well as enzyme antioxidant activity and redox reaction intermediates. Hcy levels in blood and urine have been observed to be higher in children with ASDs in prior research (125, 134, 227). Hcy levels in urine and blood of autistic people appear to be the same, implying that changes in Hcy levels in the urine may reflect changes in the blood while collecting urine samples is non-invasive, safe, and easy.

## ASD Treatment and Oxidative Stress

To learn more about the pathophysiology and diagnostic biomarkers of ASD, researchers are currently studying effective drugs and treatments (228). Increased oxidative stress is a common feature in ASD individuals, despite the fact that ASD is heterogeneous. Intervening and treating oxidative stress is one of the most effective techniques for improving the pathogenetic status of ASD patients. Therefore, various antioxidants, including sulforaphane (229), resveratrol (230–233), N-acetylcysteine (NAC) (234, 235), hesperidin (236), flavonoid (237, 238), leptin (239), minocycline, and doxycycline (240), selenium supplements (241), docosahexaenoic acid (DHA) (242), curcumin (243), agmatine (244), and sulindac (245), etc., have been reported to be employed in ASD treatment animal model experiments. In these studies, all of these antioxidants have shown positive therapeutic effects, indicating that they could be useful in the treatment of ASD.

Antioxidant therapy for ASD has only a few clinical investigations. Sulforaphane (246), resveratrol (247), coenzyme Q10 (248), NAC (249), omega-3 fatty acids (250), arachidonic acid, and DHA (251) are some of the antioxidant supplements used in these studies to treat ASD. All of these antioxidants, with the exception of resveratrol, are beneficial. Although resveratrol plays a beneficial role in the treatment of ASD animal model, its clinical study was still in its infancy. Currently, it has only one clinical study and the result is negative (252). Interestingly, a systematic review of treatments based on antioxidants reported that NAC appears to be the most effective antioxidant therapy of ASD currently (253). Furthermore, supplementing micronutrients for redox metabolism has been demonstrated to be helpful in certain children with autism (254). Treatment of ASD patients with antioxidant-rich food, on the other hand, is also a viable option. Several studies have evaluated the effectiveness of antioxidant-rich foods including broccoli (255), camel milk (256), and dark chocolate for ASD (257).

Overall, the results of these studies are positive. But as expected, some of the treatment groups in these clinical studies showed strong individual differences, reflecting the heterogeneity of ASD. It is important to note that ROS is a general term, not a specific molecule. As mentioned above, it contains a set of molecules derived from molecular oxygen, and the chemical reactivity of various ROS molecules varies widely as far as antioxidants are concerned, there are many types of antioxidants, and their specific antioxidant functions are also different. Antioxidants always have a goal that can only handle one type of ROS and not another (46, 47, 49). The causes of oxidative stress in ASD patients may differ due to genetic differences and the diversity of antioxidant defenses against oxidative stress. Using biomarkers to determine the types of antioxidants taken by each ASD patient and then supplementing them might be more successful. Antioxidants have been demonstrated to enhance behavior in persons with ASD in numerous research, however, these effects are generally transient, and only a few studies have shown a long-term behavioral reversal in people with ASD (228). Therefore, effective biomarkers for monitoring the efficacy of antioxidative therapy in ASD patients should be considered.

Some of the antioxidants mentioned above, such as sulforaphane, resveratrol, naringenin, curcumin, and agmatine, work as both antioxidants and Nrf2 activators (252). Nrf2 is a transcription factor implicated in immunological dysregulation/inflammation, oxidative stress, and mitochondrial dysfunction. Nrf2 is generally coupled to Kelch-like ECH-associated protein 1 (Keap1) in an inactive form, and the ubiquitin-proteasome system destroys the complex, allowing cells to maintain a steady low level of Nrf2 (258). The complex dissociates when subjected to oxidative stress, and Nrf2 translocates to the nucleus. Before binding to specific DNA locus antioxidant response elements (AREs), Nrf2 will heterodimerize with Maf or Jun proteins in the nucleus (259, 260). NRF2-ARE binding can regulate the expression of hundreds of cytoprotective genes including antioxidant proteins and phase II enzymes (261). Furthermore, the Nrf2/ARE pathway interacts with the NF-κB (nuclear factor kappa-light-chain-enhancer of activated B cells) pathway. The p65 subunit of NF-κB inhibits the Nrf2/ARE pathway by depriving CREB binding protein (CBP), allowing HDAC3 to recruit to MafK and interact with Keap1 (262, 263). Alternatively, free Keap1 can inhibit the NF-κB pathway by regulating the activity of the inhibitor of nuclear factor-κB kinase subunit beta (IKK-β) (264). NF-κB is a key player in the regulation of inflammation (265), as is involved in the release of pro-inflammatory cytokines such as IL-1, IL-6, IL-12, and TNF-α (266). Several studies have also shown that Nrf2 can directly regulate the availability of mitochondrial respiratory substrates, resulting in mitochondrial depolarization, reduced ATP levels, and impaired respiratory function. Furthermore, the aforementioned negative phenomena can be reversed by activating the Nrf2 pathway (267, 268).

Moreover, when induced with lipopolysaccharide (LPS), Nrf2-deficient mice have a more pronounced release of ROS, microglial activation, and neuro-inflammatory response than normal mice (269). Some studies of BTBR mice (a model of ASD) indicated that the Nrf2 system plays an important role in the regulation of neuroinflammation and oxidative stress in the brain (229, 270). A study in monocytes from people with ASD found a positive result by regulating the Nrf2 system in an *in vitro* LPS-induced inflammatory model (271) and many other studies have reported the abnormalities of the Nrf2 system in ASD individuals (272, 273). Therefore, the Nrf2 system is one of the important ways of antioxidant therapy. A systematic review of treatments based on the Nrf2 system shows a potentially beneficial result, but also explains that these treatments still lack sufficient evidence for their efficacy and safety (252). Better design and more rigorous research are needed before the treatments can be used.

## Limitations of Current Studies and Prospects

Disease progression, including ASD, is often accompanied by dramatic changes in the levels of various proteins and metabolites. Biomarkers can be used to comprehensively monitor the physiological status of ASD patients during diagnosis, intervention, and treatment, which can aid in understanding the condition, judging the treatment strategy, and monitoring efficacy and prognosis (3, 10). To this purpose, the efficacy of biomarkers and biological detection systems must meet stringent requirements. Traditional detection methods, such as Western blot analysis and enzyme-linked immunosorbent assay (ELISA), can not detect many markers at once, making comprehensive control difficult. The sensitivity of biological detection technology has improved, and more detection scenarios have been implemented, allowing for more comprehensive monitoring. Mass spectrometry has seen tremendous advancements in recent years, particularly in terms of reproducibility, performance, resolution, precision, and analytical quality.

Currently, mass spectrometry-based targeted proteomic or metabolomic approaches can effectively monitor multiple disease markers simultaneously (3, 10). Several targeted metabolomic techniques to oxidative stress markers have been developed (274) and utilized (275). Methionine, homocysteine, vitamins B6, B12, B9, and their metabolites have been accurately measured in several matrices, including breast milk, plasma, and neonatal mouse brain, using a novel approach (274). Concomitant vitamin B6, B9, and B12 deficits, as well as lower levels of methionine, GSH, SAM, and a lower SAM/SAH ratio, as well as Hcy, SAH, and 5-methyltetrahydrofolate (5-methyltetrahydrofuran) in children's urine samples, have all been linked to autism (275).

At the same time, because the brain is a part of the central nervous system and is susceptible to oxidative stress, numerous physiological abnormalities generated by oxidative stress in the brain would play a role in the development of autism. For the diagnosis and treatment of ASD, identifying the oxidative stress that occurs in the peripheral or brain is beneficial. Brain-derived neurotrophic factor (BDNF) (276), brain-derived exosomes (277), and other plasma brain-derived ASD biomarkers, have been discovered in numerous research. However, though some studies also reported some plasma biomarkers for brain oxidative damage by analyzing different kinds of samples including F4-Neuroprostanes and F2-Dihomo-Isoprostanes (278), biomarkers in peripheral body fluid samples are still insufficient to identify brain or peripheral oxidative stress at the moment. Although cerebrospinal fluid (CSF) samples can detect oxidative stress in the brain, they are not appropriate for patients with ASD due to the risk of injury during the sampling process. Because of their ease of collection, peripheral bodily fluid samples are always the best option.

Furthermore, little research has been done on peripheral blood cells, which play an important role in the immune system. Since a relationship has been demonstrated between oxidative stress and systemic inflammation (82, 83), peripheral oxidative stress and inflammation in ASD patients cannot be ignored. There is a lot of evidence that peripheral immune cells like T cells and B cells can affect brain neurons and can contribute to brain inflammation in some neural diseases (279).

With significant advances in biomedical detection technology, the limitations and defects of previous studies will be improved. Otherwise, some significant topics closely related to oxidative stress of ASD such as brain-derived factors and peripheral blood cells are worthy and promising.

## Conclusion

Many studies have demonstrated that oxidative stress plays a crucial part in the disease process of ASD because ASD cases have greater levels of oxidative stress and decreased antioxidant capability. The active use of biomarkers to monitor ASD patients' physiological status is helpful for disease diagnosis, intervention, and treatment. We mainly summarize the most recent research progress in the field of ASD oxidative stress biomarkers in this review. Many possible oxidative stress markers have been discovered in ASD, however, attempts to monitor the oxidative stress status of children with ASD are still difficult to meet clinical application standards, and additional study is needed. At the same time, we present a review of recent studies on antioxidant interventions. Several clinical investigations have found significant individual differences in some therapy groups, indicating that ASD is heterogeneous. With the development of mass spectrometry technology, mass spectrometry-based proteomics, and metabolomic methods have gradually become powerful tools for exploring biomarkers. These methods make ASD biomarker research easier and help to expand the depth and breadth of biomarker research.

## Author Contributions

XL and LS contributed to conception and design of the study. JL, HZ, JZ and XT collected related literature and tabulated it. XL wrote the first draft of the manuscript. XL, NK, XC and LS wrote sections of the manuscript. All authors contributed to manuscript revision, read, and approved the submitted version.

## Funding

This article was supported by the National Natural Science Foundation of China (Grant No. 31870825), the Shenzhen Bureau of Science, Technology, and Information (Grant Nos. JCYJ20170412110026229 and JCYJ20200812122708001), and the Shenzhen-Hong Kong Institute of Brain Science-Shenzhen Fundamental Research Institutions (Grant Nos. 2019SHIBS0003 and 2021SHIBS0003).

## Conflict of Interest

The authors declare that the research was conducted in the absence of any commercial or financial relationships that could be construed as a potential conflict of interest.

## Publisher's Note

All claims expressed in this article are solely those of the authors and do not necessarily represent those of their affiliated organizations, or those of the publisher, the editors and the reviewers. Any product that may be evaluated in this article, or claim that may be made by its manufacturer, is not guaranteed or endorsed by the publisher.
